# An electrochemical sensor for nanomolar detection of caffeine based on nicotinic acid hydrazide anchored on graphene oxide (NAHGO)

**DOI:** 10.1038/s41598-021-89427-6

**Published:** 2021-06-03

**Authors:** Jemini Jose, Viswanathan Subramanian, Sadasivan Shaji, P. B. Sreeja

**Affiliations:** 1 Department of Chemistry, CHRIST (Deemed to be University), Bengaluru, Karnataka 560029 India; 2 Facultad de Ingeniería Mecánica y Eléctrica, Universidad Autónoma de Nuevo León, 66455 San Nicolás de los Garza, Nuevo León Mexico; 3 Department of Industrial Chemistry, Alagappa University, Karaikudi, Tamil Nadu 630003 India

**Keywords:** Materials science, Graphene

## Abstract

A simple modified sensor was developed with nicotinic acid hydrazide anchored on graphene oxide (NAHGO), by ultrasonic-assisted chemical route, using hydroxy benzotriazole as a mediator. Structural and morphologies of NAHGO samples were investigated in detail by Fourier-Transform Infrared spectroscopy (FT-IR), Powder X-ray diffraction (P-XRD), Raman spectroscopy, Scanning electron microscopy (SEM), X-ray photoelectron spectroscopy (XPS), and Thermogravimetric analysis (TGA). The detailed morphological examination and electrochemical studies revealed the delaminated sheet with the tube-like structure of NAHGO provided the route for more electroactive surface which influenced the electrooxidation of caffeine with increased current. The electrochemical behaviour of NAHGO on a glassy carbon electrode (GCE) for caffeine detection was demonstrated by employing voltammetric techniques. The influence of scan rate, pH, and concentration on caffeine's peak current was also studied. The NAHGO sensor was employed for the determination of caffeine in imol plus and energy drinks. The detection limit determined was 8.7 × 10^–9^ M, and the best value was reported so far. The results show that NAHGO modified electrodes are one of the best preferences to establish new, efficient, and reliable analytical tools for the detection of caffeine.

## Introduction

Carbon materials have a pivotal role in detecting an unsafe amount of organic and inorganic molecules due to their highly porous structure with large surface area and excellent electronic properties^1^. Graphene oxide (GO) has received much attention among carbon nanomaterials due to its high mechanical strength, thermal, and electrical conductivity^2^. Besides, the carboxyl, hydroxy, and epoxy groups create GO as a desirable platform for further functionalizing molecules^3,4^. Other than functionalization approaches of GO, heteroatom doping is also important since it modifies the properties of GO extensively. Among various heteroatom doping, nitrogen doping is widely utilized to employ doping of GO. This is because of the easiness of manipulating GO to improve more active sites for fuel cell, biosensing, adsorption, supercapacitor, and solar cell applications^5,6^. Nitrogen doping of GO creates different kinds of nitrogen such as pyrrolic-N, pyridinic-N, and graphitic-N in the GO lattice, which enrich the electronic properties of GO^7,8^. It has been reported that nitrogen doping changes the spin density and charge distribution on the surface of GO, with the strong interaction between the π-system of GO and the lone pair of electrons of nitrogen^9^. At present, different methods such as plasma treatment, segregation growth, chemical vapour deposition, and arc discharge method have been developed for the synthesis of nitrogen-doped GO. However, the practical applications of these methods are limited due to high and complex reaction temperature conditions^6^. Hence simple organic compounds with amino groups like urea, melamine, glycine, and hydroxyl amine are utilized to achieve nitrogen doping. The integration of nitrogen atoms in the GO framework brings out significant changes in the electronic structure resulting in the reduction of GO^10^. The incoming molecules' active sites such as diisopropylamine, diaminopyridine, dibenzylamine, phenylenediamine, piperidine, phenylethylamine, 2,4-dinitrophenylhydrazine, Bis-(pththalimidoethyl)-amine, and Tris-(hydroxymethyl) methyl aminoethane generate new covalent bonds with GO^11,12^. Therefore, in this paper we report the synthesis of NAHGO by a simple and cost effective ultrasonic-assisted chemical route where the amino group of NAH reacts with the oxygen containing carboxylic acid, hydroxyl, and epoxy groups of GO that brings the nitrogen doping and the simultaneous reduction of GO.

Caffeine (1,3,7-trimethyl xanthine) is one of the most consumed purine alkaloid ingredients. It is a central nervous system stimulant, fatigue alleviator, sharpener of mental function and concentration, taste refresher with a unique taste, antioxidant, booster of metabolism, and associated health benefits^13,14^. It is commonly found in coffee beans, tea leaves, guarana berries, pharmaceutical drugs, processed foods, and energy drinks^15^. The sensible amount of caffeine in medications reduces constipation, depression, and headache. Like all other drugs, caffeine overdose produces anxiety, heartburns, increased blood pressure, seizures, bone mass loss, and cardiovascular diseases^16,17^. On the other hand, the extracts of caffeine act as a pesticide for plants, and it has been considered an indicator for water contamination in sewers^18,19^. Hence, there is a need for detecting caffeine reliably at lower concentrations, especially in common beverages, caffeinated food, and drugs, since the average consumption of caffeine per day is up to 0.4 g^20^.

Different analytical techniques are currently available to determine caffeine, such as high-performance liquid chromatography (HPLC), UV spectrometry, capillary electrophoresis, spectrophotometry^21–24^. These analyses are complicated because of the lengthy and time-consuming procedures. Hence the development of highly sensitive, consistent, and fast methods is essential.

The electrochemical method has proved its quality and efficiency for the fabrication of sensing devices^25,26^. It acts as a powerful tool for detecting and monitoring the unsafe amount of organic and inorganic compounds using different carbon materials. Caffeine has already been electrochemically detected with the boron-doped diamond electrode, DNA-functionalised single-walled carbon nanotubes (SWCNT), attapulgite/nafion, gold nanoparticle, gold-chitosan, multiwalled carbon nanotubes (MWCNT)/diamond-like carbon films, glutathione-rGO, etc^27–31^. Hence, we propose a new kind of electrochemical sensor supported by GO and nicotinic acid hydrazide (NAH) to detect caffeine. The synthesised NAHGO was analysed by different spectroscopic and morphological methods and then studied caffeine’ electrochemical detection. We found that NAHGO showed catalytic activity for the electro-chemical oxidation of caffeine.

## Results and discussion

### Characterizations of NAHGO

**Figure 1:**
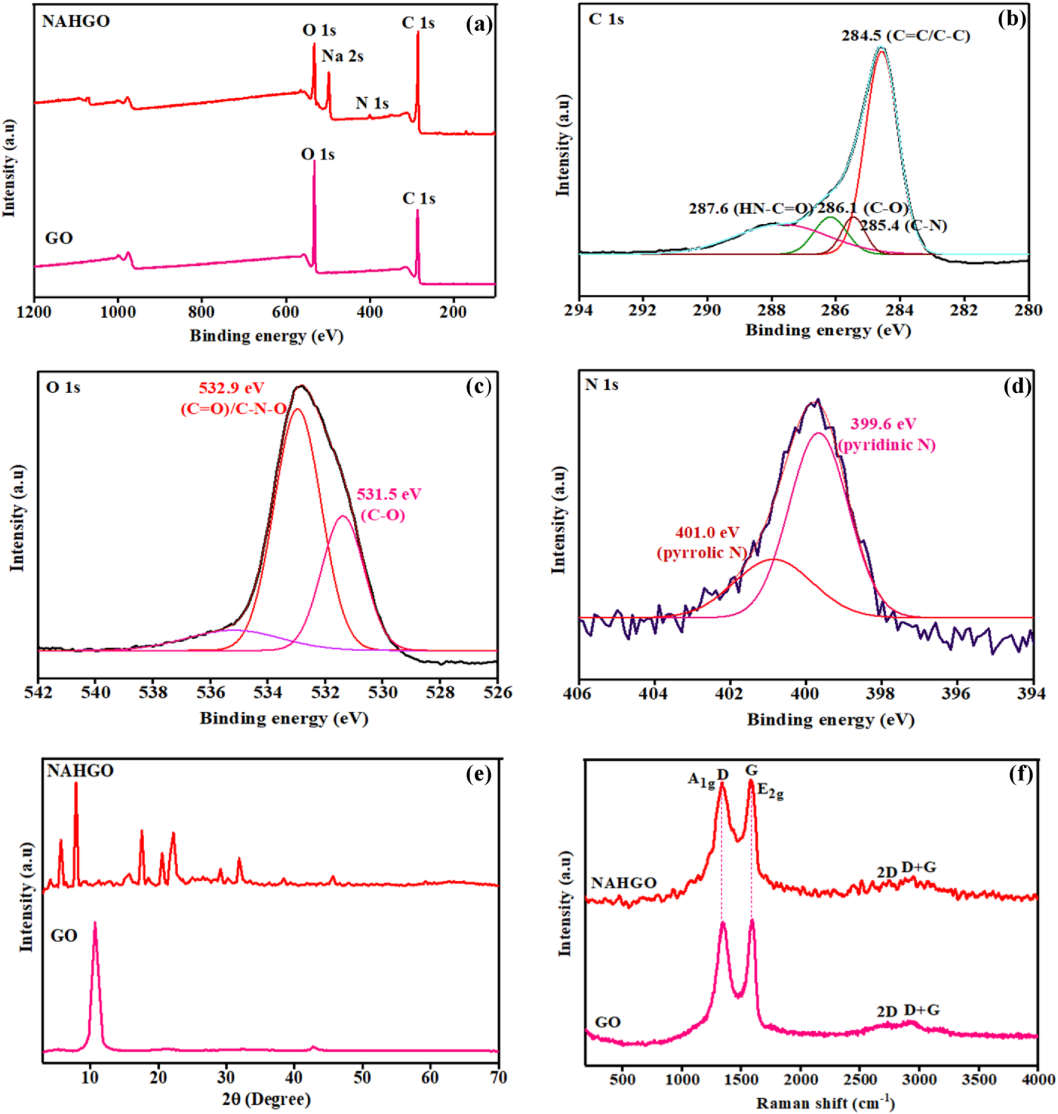
(**a**) XPS wide scan spectra of GO and NAHGO, deconvoluted peaks of (**b**) C1s, (**c**) O1s, and (**d**) N1s (**e**) Powder XRD patterns, and (**f**) Raman spectra of GO and NAHGO.

The X-ray photoelectron spectrum of the hybrid NAHGO is performed to assess the surface composition and chemical states of the elements present in NAHGO. The broad scan spectrum of GO and NAHGO are displayed in Fig. 1a. GO shows the peaks of C1s and O1s, whereas NAHGO exhibits carbon, nitrogen, and oxygen, with major prominent peaks at 285.12, 401.23, and 533.41 eV corresponds to C1s, N1s, and O1s, respectively. The C1s high-resolution spectrum is deconvoluted into four components, as shown in Fig. 1b. The peaks at 284.5, 285.4, 286.1, and 287.6 eV attributed to C–C/C=C of graphene oxide framework, C–N, C–O, and HN–C=O bonds. The deconvoluted peaks of O1s at 531.5 and 532.9 eV, as indicated in Fig. 1c, are due to the characteristic C–O, C=O/C–N–O of NAHGO^32,33^. The individual peaks of N1s at 399.6 and 400.8 eV, which are exhibited in Fig. 1d, attribute to the presence of pyridinic N and pyrrolic N, respectively. There is a high peak at 500 eV due to the auger line of sodium (Na 2 s or Na_KLL_)^33,34^. The EDAX data is shown in Fig. S1. The XRD patterns of GO and NAHGO is presented in Fig. 1e. The intense 2θ peak at 10.7° (001) diffraction plane of as prepared GO shows an inter-planar distance of 0.83 nm, calculated by Bragg’s equation. In NAHGO, 10.7° of GO is disappeared and shifted to a high 2θ value, showing the reduction of oxygen containing functional groups and incorporating nitrogen atoms onto graphene surface (XRD of NAH is shown in Fig. S2). The Raman spectra of GO and NAHGO display two major bands, as shown in Fig. 1f**.** The C=C stretching vibration of sp^2^ hybridised carbon systems illustrate the G band at about 1592 cm^−1^ and D band at 1352 cm^−1^ corresponds to the distortions in the edge-centred band structure of the carbon framework of GO^35^. In NAHGO, these two peaks shift to 1581 cm^−1^ and 1341 cm^−1^, confirming the reduction in hybrid GO^36^. The intensity ratio I_D_/I_G_ of GO and NAHGO are 0.96 and 1.03, respectively. The increased intensity ratio of I_D_ /I_G_ in NAHGO attributed to defects present after the functionalization of GO^37^.

**Figure 2:**
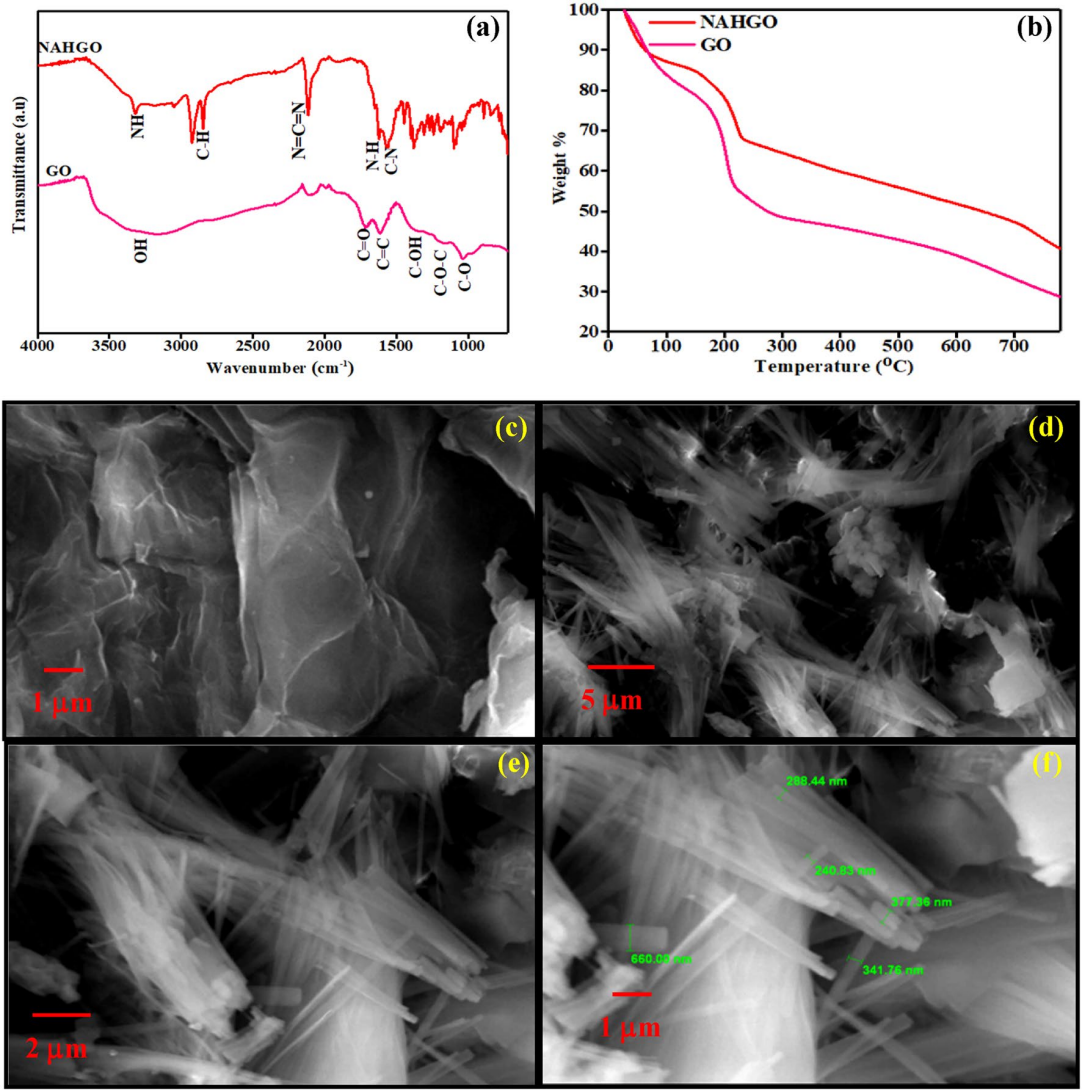
(**a**) FTIR spectra, (**b**) Thermogravimetric analysis, (**c**) SEM images of GO, and (**d**–**f**) NAHGO in different magnifications.

The IR spectra of GO and NAHGO are shown in Fig. 2a**.** The broad peak at 3375 cm^−1^ is attributed to the stretching vibration of carboxyl –OH for GO. The C=O, C–OH, and C–O of carboxylic and epoxy groups represent peaks at 1721 cm^−1^, 1215 cm^−1^, and 1030 cm^−1^, respectively^37–39^. Three new peaks are observed in the spectra of NAHGO. They are at 1625 cm^−1^, correspond to the amide carbonyl stretch; 1561 cm^−1^ is due to the N–H of amide band; 1445 cm^−1^, the C–N stretching of amide bond^40,41^. This is in accordance with XPS, support the nitrogen doping of GO by nicotinic acid hydrazide. The absorption bands at 2950 and 2900 cm^−1^ are assigned to the symmetric and asymmetric stretching of C–H vibrations^41^. The band at 2100 cm^−1^ corresponds to N=C=N vibrations^42,43^. Thermal stability of the GO and NAHGO were investigated by TGA and are shown in Fig. 2b. The slow degradation of NAHGO with GO indicates the high stability which is acquired by reducing GO and the presence of nitrogen atoms in NAHGO. The XPS data of GO and NAHGO also supports the same. The GO and NAHGO were examined by SEM to understand more about these compounds' morphology and microstructure. As seen in Fig. 2c–f, GO demonstrated the extended sheet-like structures, and NAHGO results the deformation of GO owing to the amide bond formation and esterification reaction with NAH. In NAHGO, the small tube-like structures of 245–342 nm are distributed among the layered sheets of GO. The formation of needle like morphology is likely due to the distortion of sheets or to the impurities in the sample. The incorporation of nitrogen moieties delaminates the sheet structure with significant disorder in the graphitic structure of GO. The reduction and the integration of nitrogen in GO enhance ion transport, suitable for electrochemical sensing applications.

### Electrochemical activity of NAHGO

**Figure 3:**
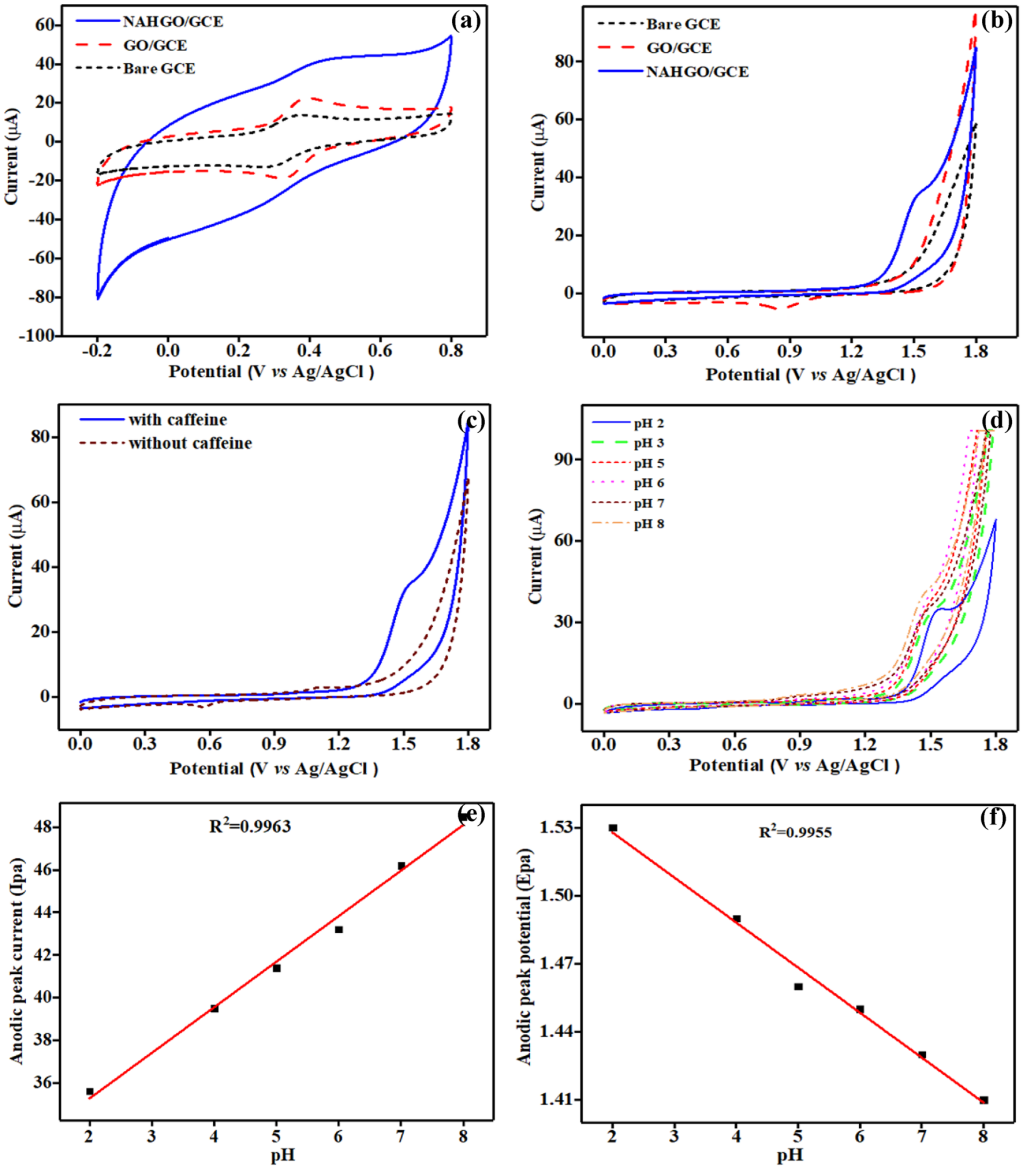
Cyclic voltammograms of the bare GCE, GO, and NAHGO (**a**) in the presence of 0.001 M K_4_Fe(CN)_6_ in 0.1 M KCl, (**b**) at pH 2 with 100 × 10^–6^ M of caffeine at a scan rate of 50 mV s^−1^, Cyclic voltammograms of NAHGO (**c**) with and without caffeine, (**d**) at pH values of 2, 3, 5, 6, 7, 8, in 100 × 10^–6^ M of caffeine, at a scan rate of 50 mVs^−1^, Calibration plot of (**e**) pH vs. anodic current (Ipa), and (**f**) pH vs. anodic peak potential (Epa).

The surface area of GO and NAHGO modified working electrodes were probed using 0.001 M K_4_Fe(CN)_6_ in 0.1 M KCl at a scan rate of 50 mV s^−1^ in a potential window of − 0.2 to 0.8 V, and are shown in Fig. 3a. The poor electrochemical response of K_4_Fe(CN)_6_ in 0.1 M KCl at bare GCE implies a slow transfer of electrons at the surface. The slight increase of redox peak currents at GCE modified NAHGO than GO, possibly due to fast electron transfer and the large electroactive surface area at NAHGO. This is effective in offering more active sites for caffeine oxidation. The scan rates of NAHGO in a mixture of 0.001 M K_4_Fe(CN)_6_ and 0.1 M KCl and plots of anodic current (Ipa) and cathodic current (Ipc) *vs.* square root of the scan rate (υ^1/2^(mVs^−1^)^1/2^ (Figs. S3 and S4a,b) were observed. The electrochemically active surface areas of all the GCE modified electrodes were calculated with the individual slopes of the plot using *Randles Sevick equation*

1 $${\text{I}}_{{\text{p}}} = ({2}.{69} \times {1}0^{{5}} ){\text{ n}}^{{{3}/{2}}} {\text{D}}^{{{1}/{2}}} \upsilon^{{{1}/{2}}} {\text{AC}}^{*}$$

which relates the peak current of the active species with the surface area for a reversible process^44^. I_p_ refers to the anodic peak current, n is the total number of electrons transferred (n = 1), A is the effective surface area of the electrode, D is the diffusion coefficient of K_4_Fe(CN)_6_, C^*^ is the concentration of K_4_Fe(CN)_6,_ and υ is the scan rate. The calculated electroactive surface areas for bare GCE, GO, and NAHGO modified electrodes are 0.078 cm^2^, 0.126 cm^2^, and 0.29 cm^2^, respectively. NAHGO seemed to provide an enhanced active surface area, presumably owing to the defects in GO with electron-rich nitrogen moieties^45^.

### Electrocatalytic characteristics of NAHGO for the oxidation of caffeine

The electrochemical activities of bare GCE, GO, and NAHGO were further investigated in the presence of caffeine. Figure 3b shows CVs of bare GCE, GO, and NAHGO modified GCE electrodes, at pH 2 with 100 × 10^–6^ M of caffeine, at a scan rate of 50 mV s^−1^. The electrocatalytic behaviour of the NAHGO was also examined in the absence and presence of caffeine. It is shown in Fig. 3c. The caffeine oxidation peak in 1.5 V indicates the oxidation of caffeine on NAHGO modified GCE, according to the previously reported results^31,46^. Caffeine has a tertiary amine group with three alkyl groups and an amide group in which a carboxyl group is connected to a nitrogen atom. In addition to these groups, imine, carboxyl, methyl, and alkene groups are also present in caffeine. The carbon–oxygen and carbon–nitrogen covalent bonds make caffeine more polar due to its molecular geometry. The distinct positive and negative parts created by the overall molecular dipole of caffeine have a strong affinity to the ends of other oppositely charged ends of the NAHGO by intermolecular attractions. Though the three nitrogen atoms of caffeine are methylated (N1, N3, and N7), as seen in Fig. S5, caffeine serves as a hydrogen bond acceptor with the ring nitrogen atom (N9) and carbonyl oxygen atoms (O2 and O6)^47^. Therefore the interaction of hydrogen is highly dominated by these positions of caffeine. The effective probes of interactions in NAHGO are epoxide, alcohol, carboxylic acid, pyrrolic, and pyridinic nitrogen moieties^48^. The alcohol and carboxylic acid oxygen containing functional groups and the NH of pyrrolic nitrogen can act as hydrogen bond donors and acceptors. In this case, they play the role of hydrogen donor and make electrostatic interactions with caffeine. Hence, the increased electrochemical response of NAHGO arises from the efficient interactions of highly polar functional groups of caffeine and NAHGO, which generate charge accumulations on highly polar functional groups turned out to create electrostatic interactions. These interactions are less in GO due to the fewer heteroatoms, which leads to not as much current response of NAHGO.

### Effect of supporting electrolytes

**Figure 4:**
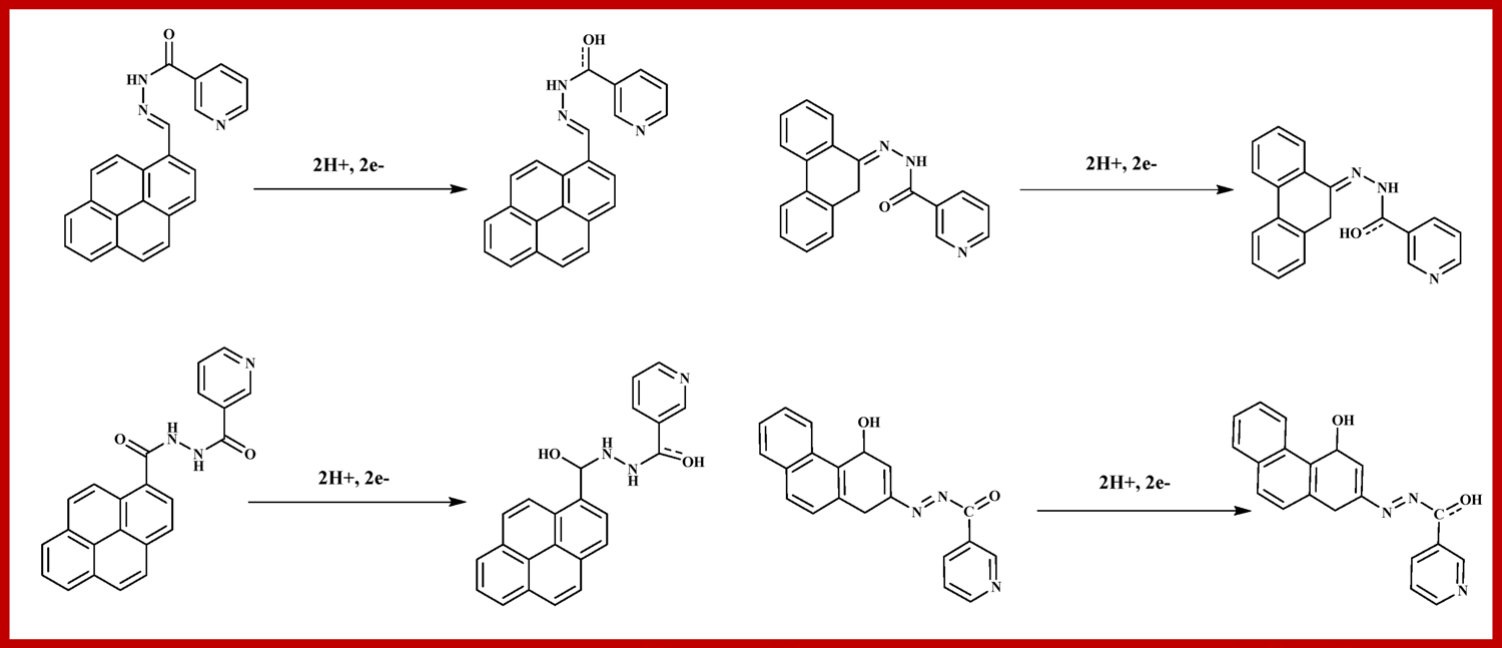
The interactions of NAHGO at pH 2.

Different electrolyte solutions of pH were used to understand the effect of supporting electrolyte on the cyclic voltammetric behaviour of caffeine. The effect of pH on the peak currents and peak potentials of caffeine electro-oxidation (100 × 10^–6^ M) by NAHGO electrode was studied and is represented in Fig. 3d. Though pH 8 has a higher peak current and lower anodic peak potential for the caffeine oxidation at pH 8, the oxidation peak current is not as sharp as pH 2. It is also reported earlier that pH 2 (acidic medium) is best to occur the oxidation of caffeine^27,28,30^. So, further electrochemical studies are performed at pH 2. The linear dependence of anodic peak current and anodic peak potential with pH for caffeine is shown in Fig. 3e,f. The corresponding regression equations are: Ipa = 0.214pH + 0.307 (R = 0.9963) and Epa = 2.56–0.0491 pH (R = 0.9955). This implies that the electrochemical oxidation occurs with an equal number of proton and electron transfer process^49^. In the presence of acidic pH, the exchange of electrons between the π bonds of the graphene sheet and the lone pair of electrons bring the polarization effect at the electrode surface. The interactions of NAHGO with acidic protons are shown in Fig. 4.

### Effect of scan rate

**Figure 5:**
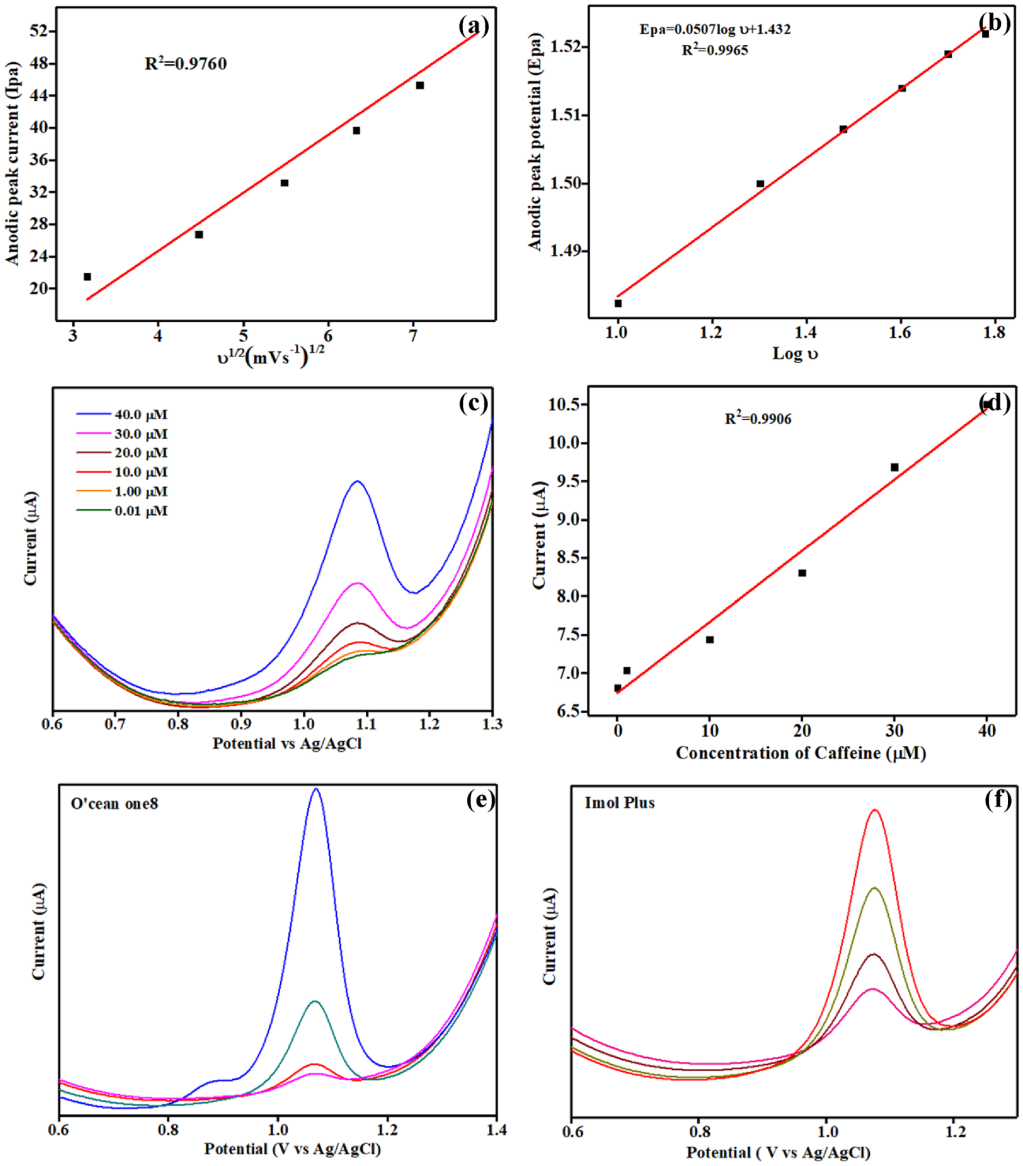
Calibration plot of (**a**) the square root of the scan rate (υ^½^) *vs.* anodic peak current (Ipa) and (**b**) logarithm of scan rate (log υ) vs. anodic peak potential (Epa), (**c**) Differential pulse voltammogram (DPV) of the NAHGO modified GCE electrodes at a pH value of 2 with standard additions of caffeine in the order 0.01 × 10^–6^ M, 1.0 × 10^–6^ M, 10 × 10^–6^ M, 20 × 10^–6^ M, 30 × 10^–6^ M, 40 × 10^–6^ M (**d**) Calibration plot of caffeine concentration (μM) vs. anodic peak current (Ipa), Differential pulse voltammogram of the NAHGO in (**e**) *O’cean one8* energy drink and (**f**) *imol plus.*

To obtain more knowledge about the adsorption properties of NAHGO, the CVs of 100 × 10^–6^ M caffeine on NAHGO at various scan rates (Fig. S6) were studied for the electro-oxidation process of caffeine. As displayed in Fig. 5a, caffeine's anodic peak current increases linearly with the increase of scan rate from 10 to 60 mV s^−1^. The linear relationship of the oxidation current and the square root of the scan rates presents that the electrochemical reactions on the proposed electrode are diffusion controlled. The oxidation peak potential (Epa) shifts to more positive potential as the gradual increase of scan rates. Laviron model was used to describe the kinetic parameters of the electrochemical oxidation of caffeine^50^. The linear relationship of anodic oxidation potential with logarithms of scan rate is illustrated by this method. According to Laviron, the correlation between the scan rate and the oxidation peak potential is explained as follows:

2 $$Epa = E^{0} + {{2.3RT} \mathord{\left/ {\vphantom {{2.3RT} {\left[ {\left( {1 - \infty } \right)nF} \right]}}} \right. \kern-\nulldelimiterspace} {\left[ {\left( {1 - \infty } \right)nF} \right]}}\$ log\;\upsilon$$

**Figure 6:**
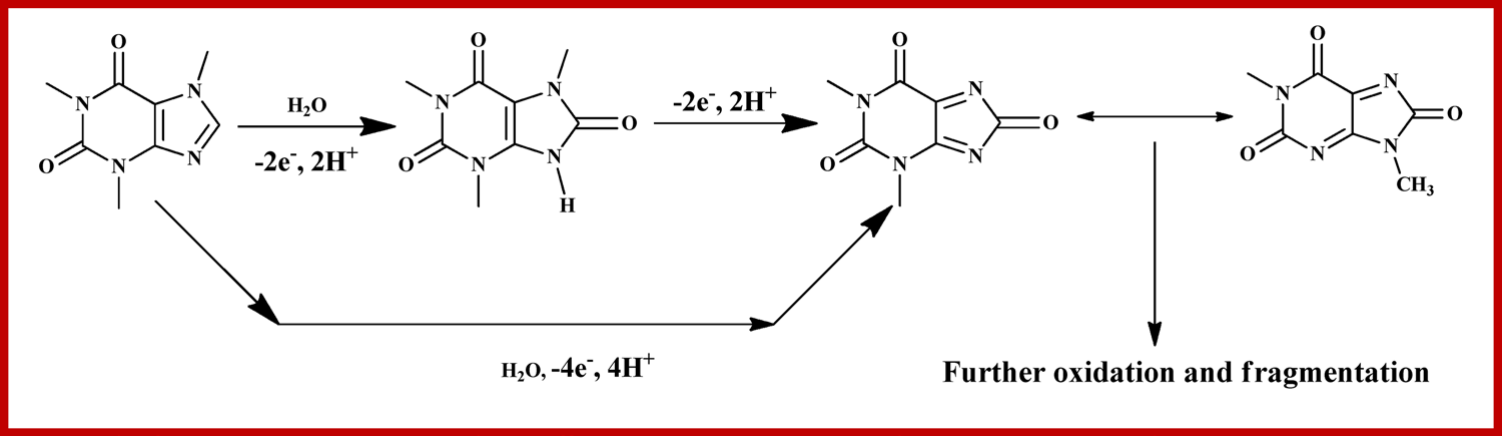
Mechanism of the electrochemical oxidation of caffeine at NAHGO.

As shown in Fig. 5b, Epa exhibits the linear dependence with logarithms of scan rate (log υ), and the equation for linearity is Epa = 1.4326 + 0.0507logυ with R^2^ = 0.9965. The electrochemical transmission coefficient of an irreversible process is about 0.4–0.6, and for those processes, the value is considered to be 0.5^51^. Accordingly, the electronic transmission number (n) of caffeine's electrochemical oxidation is calculated as 3.85, which is in good agreement with the previous results^52^. The mechanism of electrochemical oxidation of caffeine is shown in Fig. 6, as reported earlier^53^. Substituted uric acid is formed in the first step, by the oxidation of two electrons and two protons at C-8-N-9 position, followed by the formation of 4,5 diol of uric acid analogue through the primary two electrons and two proton oxidation.

### Analytical performance

The differential pulse voltammetric method was found to be a more sensitive method in the present work, as it produced better electrochemical performance indicators than the CV technique. Therefore, in order to achieve the favourable analytical performance using the differential pulse voltammetric (DPV) method, the parameters such as pulse amplitude (from 0.05 to 0.15 V), pulse width (from 0.005 to 0.5 s), and pulse period (0.1–3 s) is optimised. The best oxidation peak of caffeine was recorded as follows: pulse amplitude 0.05 V, pulse width 0.06 s, and pulse period of 0.5 s with NAHGO modified electrode in 0.1 M H_2_SO_4_ at pH 2 in caffeine. Different concentrations from 0.01 to 40 of caffeine solutions were prepared to examine the relationship between the peak current and caffeine concentration. Figure 5c displays the linear increase of the anodic peak current (Ipa) with concentrations of caffeine. The calibration graph (Fig. 5d) for caffeine at NAHGO obeys the linear regression equation: Ipa (μA) = 6.883 + 0.0989C; R^2^ = 0.9906. The limit of detection (LOD) of caffeine is calculated to estimate this method for the determination of caffeine using the formula 3.3σ/S, where σ represents residual standard deviation and S is the slope of the calibration curve^54^ and the LOD for NAHGO modified electrode is found to be 8.7 × 10^–9^ M, the best reported so far. Moreover, it is compared with other reported values for the determination of caffeine using electrochemical techniques. They are presented in Table 1 and which shows the effectiveness of the proposed NAHGO sensor.

**Table 1:** An overview of electrochemical methods for the determination of caffeine.

Modified electrodes	Technique	Linear range (10^–6^ M)	Detection limit (10^–6^ M)	References
BQ/CPE	SWV	500–8000	51.00	^54^
Bi-CNT/CPE	SWV	51.03–1026	0.182	^55^
DNA/CNT/CPE	SWV	0.5120–61.70	0.350	^56^
MIP/CPE	DPV	0.0600–25.00	0.015	^57^
CTAB/GR/GCE	DPV	0.300–100.0	0.091	^58^
SWCNT/CPE	DPV	0.250–100.0	0.120	^59^
PT/TiO_2_-GR/GCE	DPV	25.00–200.0	0.500	^60^
Nitrogen doped graphene	SWV	0.060–50.00	0.020	^61^
Poly(Alizarin Red S)/GCE	SWV	0.500–250.0	0.060	^62^
Attapulgite/Nafion/GCE	DPV	1.000–4.000	0.040	^28^
NAHGO/GCE	DPV	0.010–40.00	0.008	This work

### Determination of caffeine in an energy drink and pharmaceutical sample

Finally, to estimate the practical application of this method, NAHGO/GCE was used to determine caffeine in imol plus and O’cean one8 energy drink. The energy drink was degasified by ultrasonication for 10 min before the analysis and diluted with supporting electrolyte (100 × 10^–6^ M). The tablet was grounded with mortar to a fine powder, dissolved in supporting electrolyte (100 × 10^−6^ M), and sonicated for 10 min. Subsequently, the appropriate amount of the resulting dispersion was selected and centrifuged. 9 cm^3^ of 0.1 mol L^−1^ pH 2 solution was added to 1 cm^3^ of the sample solution, and recorded the DPVs (Fig. 5e,f). The mean time to calculate the recovery standard solutions of caffeine is added and represented in Table S1. The recovery range of 94.9–101.9% suggests that NAHGO is adequate for practical sensing applications.

## Experimental

### Materials

Graphite, potassium permanganate, sulphuric acid (36 M), hydrochloric acid (5%), sodium hydroxide, hydrogen peroxide (30%), hydroxy benzotriazole, dicyclohexyl carbodiimide, nicotinic acid hydrazide, potassium chloride, potassium ferrocyanide, citric acid, sodium citrate, caffeine powder, potassium dihydrogen phosphate, dipotassium hydrogen phosphate were purchased from Sigma Aldrich. All the reactions were carried out under a nitrogen atmosphere with the use of the standard Schlenk technique. All the solvents and reagents were of analytical grade and used without further purification. The O’cean one8 energy drink and imol plus were purchased from a local market and pharmaceuticals, respectively. The stock solutions of caffeine (100 × 10^–6^ M) were prepared fresh each time of experiments. Aqueous solutions of different pH of buffers were prepared, diluted using millipore water.

### Synthesis of nicotinic acid hydrazide anchored graphene oxide (NAHGO)

**Figure 7:**
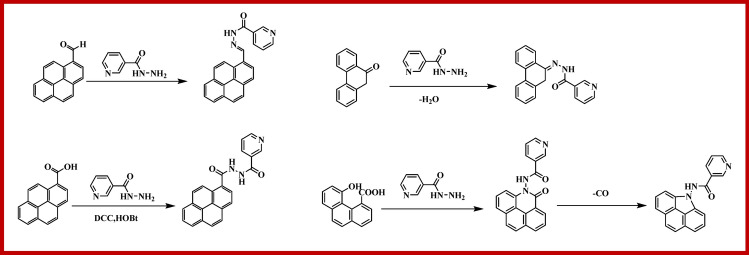
Reactions occur during the synthesis of NAHGO.

Graphene oxide (GO) was synthesised from graphite by modified Hummers method^63^. The nicotinic acid hydrazide anchored graphene oxide (NAHGO) was synthesised by the dispersion of GO (0.2 g) in 20 cm^3^ dimethylformamide (DMF) by ultrasonication method. To the solution, sodium hydroxide pellets (0.2 g, 5.0 mmol) were added, and the resulting solution was stirred for 60 min at room temperature. Consequently, nicotinic acid hydrazide (0.2 g, 1.45 mmol), hydroxybenzotriazole (0.2 g, 2.81 mmol) followed by dicyclohexylcarbodiimide (0.2 g, 2.66 mmol) were added to the above reaction mixture and stirred for 24 h in nitrogen atmosphere. The resultant NAHGO powder collected by centrifugation was added to pure DMF and the resulting suspension was again centrifuged to remove the side products. This process was repeated twice with DMF and then with water to remove DMF to give pure NAHGO. The NAHGO prepared was dried overnight at 60 °C. The amide, amine, and pyridinic groups are present in NAHGO. The reaction of the amino group of NAH with the hydroxyl of carboxylic acid generates another amide bond in NAHGO. The imine can form with the amine group of NAH and the –C=O or –HC=O group of GO. The nearby carboxylic acid and hydroxyl groups can undergo an esterification reaction followed by the generation of a pyrrole ring. Hence we believe that the treatment of NAH in GO creates more defects due to the reduction of GO with nitrogen doping. The suggested reactions which occur during the synthesis of NAHGO are shown in Fig. 7.

### Characterisation techniques

**Figure 8:**
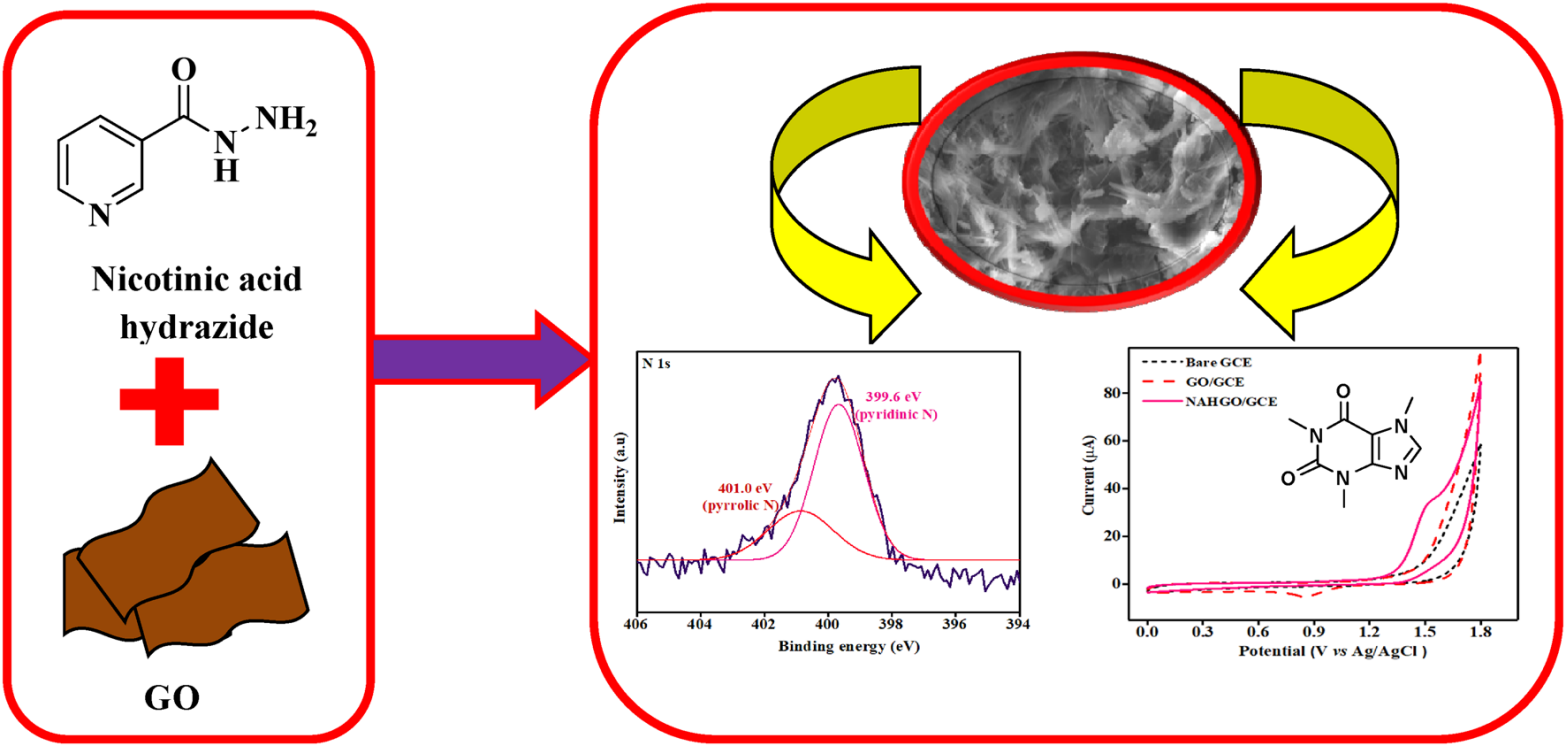
Synthesis and electrochemical activity of NAHGO as a sensor.

The characteristic functional groups of different samples were recorded using an Agilent technologies FTIR spectrometer in the range 400–4000 cm^−1^. Powder X-ray Diffraction (P-XRD) patterns were carried out in a Brucker AXSD8 Advance using Ni-filtered Cu-K_α_ X-ray source (λ = 1.5406 Å), with a scan speed of 2° min^−1^. Thermogravimetric analysis (TGA) was recorded in Perkin Elmer STA 6000. The composition and disorders of anchored compounds were analyzed by X-ray photoelectron spectroscopy (Thermoscientific K-alpha surface analyser). Raman spectra of GO and NAHGO were recorded in HORIBA Jobin Yvon Lab RAM HR 800, equipped with a thermoelectrically cooled CCD detector. The morphology and size of GO and NAHGO were recorded on a JEOL Model JSM-6390LV scanning electron microscope. Cyclic voltammetric (CV) and differential pulse voltammetric (DPV) measurements were carried out on a CHI608E electrochemical workstation with a three-electrode system saturated Ag/AgCl acted as the reference electrode, Pt wire as a counter electrode, and the modified glassy carbon electrode as the working electrode. The preparation of the NAHGO modified glassy carbon electrode and the different buffers used are given in supporting information. Synthesis and electrochemical activity of NAHGO as a sensor are shown in Fig. 8.

## Conclusion

In summary, the synthesis and properties of NAHGO for electrochemical detection of caffeine were discussed. The structure and morphologies of NAHGO were studied by IR, P-XRD, Raman, SEM, and TGA. The electrochemical oxidation of NAHGO towards the alkaloid caffeine was analyzed. NAHGO displayed enhanced electrochemical activity towards the determination of caffeine, with an improved detection limit of 8.7 × 10^–9^ M. The NAHGO sensor was also employed to determine caffeine in *Imol plus* and *O’cean one8* energy drink. Electrochemical studies exhibited that the NAHGO can be used as an efficient and reliable analytical tool for caffeine detection.

## Supplementary Information

Supplementary Information.
