# Supplementary material for: An electrochemical sensor for nanomolar detection of caffeine based on nicotinic acid hydrazide anchored on graphene oxide (NAHGO)
Source: Sci Rep. 2021 Jun 3;11:11662. doi: 10.1038/s41598-021-89427-6 (PMC8175555; doi:10.1038/s41598-021-89427-6)
Supplement: Supplementary file 1 — Supplementary Information. [file 41598_2021_89427_MOESM1_ESM.doc]

**Supporting information**

**An electrochemical sensor for nanomolar detection of caffeine based on nicotinic acid hydrazide anchored on graphene oxide (NAHGO)**

Jemini Jose^a^, Viswanathan Subramanian^c^, Sadasivan Shaji^b^, Sreeja P B^a*^

^a^Department of Chemistry, CHRIST (Deemed to be University), Bengaluru 560029, Karnataka, India

^b^Facultad de Ingeniería Mecánica y Eléctrica, Universidad Autónoma de Nuevo León, Nuevo León, 66455, México

^c^Department of Industrial Chemistry, Alagappa University, Karaikudi 630003, Tamil Nadu, India

^*^Corresponding author. Tel: +91 8884148191. E-mail: [sreeja.pb@christuniversity.in](mailto:sreeja.pb@christuniversity.in)

**Preparation of NAHGO modified glassy carbon electrodes**

Simple casting method was used to fabricate the NAHGO modified glassy carbon electrode (GCE). Before the modification of GCE, it was polished with 1 micron and 0.05 micron alumina slurries and followed by ultrasonication in acetone, ethanol and millipore water. The cleaned bare GCE was dried at room temperature. Then aliquots of 7 μL of NAHGO/ethanol dispersion was drop casted into the GCE surface and dried in air for the preparation of NAHGO modified glassy carbon electrode.

**Buffers**

The compounds used for buffer preparation for the determination of caffeine are:

pH 2 hydrochloric acid and potassium chloride

pH 3-6 citric acid and sodium citrate

pH 7-8 monopotassium phosphate and dipotassium phosphate

EDAX spectra of GO and NAHGO

| GO  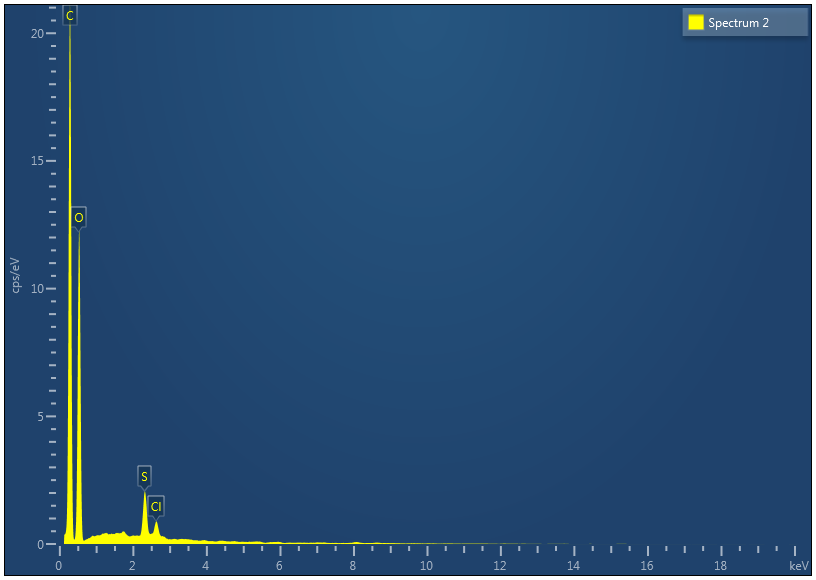  **a**   | Element | Line Type | Wt% | Atomic % | | --- | --- | --- | --- | | C | K series | 55.34 | 62.81 | | O | K series | 42.71 | 36.39 | | S | K series | 1.41 | 0.6 | | Cl | K series | 0.54 | 0.21 | | Total: |  | 100 | 100 | | NAHGO  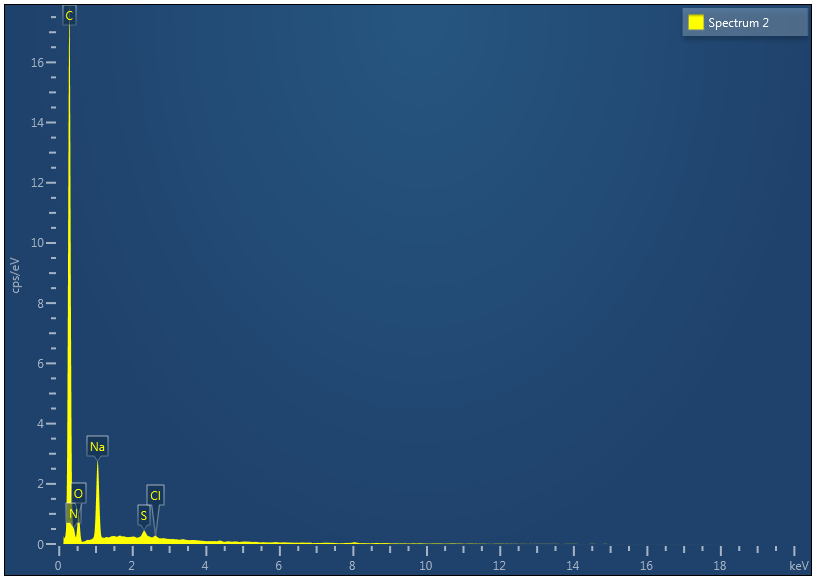  **b**   | Element | Line Type | Wt% | Atomic % | | --- | --- | --- | --- | | C | K series | 78.64 | 84.03 | | N | K series | 3.44 | 3.15 | | O | K series | 11.92 | 9.56 | | Na | K series | 5.48 | 3.06 | | S | K series | 0.34 | 0.14 | | Cl | K series | 0.17 | 0.06 | | Total: |  | 100 | 100 | |
| --- | --- | --- | --- | --- | --- | --- | --- | --- | --- | --- | --- | --- | --- | --- | --- | --- | --- | --- | --- | --- | --- | --- | --- | --- | --- | --- | --- | --- | --- | --- | --- | --- | --- | --- | --- | --- | --- | --- | --- | --- | --- | --- | --- | --- | --- | --- | --- | --- | --- | --- | --- | --- | --- | --- | --- | --- | --- |

**Fig. S1** EDAX spectra of GO and NAHGO

**XRD of NAH**

**Fig.S2** XRD of NAH

**Cyclic voltammograms of NAHGO in the mixture of 0.001M K_4_Fe(CN)_6_ and 0.1 M KCl in different scan rates, and calibration graphs**

Cyclic voltammograms of NAHGO in the mixture of 0.001M K_4_Fe(CN)_6_ and 0.1 M KCl in different scan rates of 10 mV s^-1^ to 90 mV s^-1^ is shown in Fig. S3. The Plot of anodic current (Ipa) and cathodic current (Ipc) *vs* square root of the scan rate (υ^1/2^(mVs^-1^)^1/2^ is given in Fig. S4a and b.

**Fig. S3** Cyclic voltammograms of NAHGO in the mixture of 0.001M K_4_Fe(CN)_6_ and 0.1 M KCl in different scan rates of 10 mV s^-1^ to 90 mV s^-1^

**Fig. S4 (a)** Plot of anodic current (Ipa) and (c) cathodic current (Ipc) *vs* square root of the scan rate (υ^1/2^(mVs^-1^)^1/2^, **(b)** Cyclic voltammograms of NAHGO at pH of 2, with 100 $10^-6^ M of caffeine at a scan rate of 10 mV s^-1^ to 60 mV s^-1^

**Chemical structure of caffeine**

Chemical structure of caffeine is shown in Fig. S5

**Fig. S5** Chemical structure of caffeine

**Cyclic voltammograms of NAHGO at pH of 2, with 100 $10^-6^ M of caffeine at different scan rates**

Cyclic voltammograms of NAHGO at pH of 2, with 100 $10^-6^ M of caffeine at a scan rate of 10 mV s^-1^ to 60 mV s^-1^ in Fig. S6.

**Fig. S6.** Cyclic voltammograms of NAHGO in 0.1 M H_2_SO_4_ at a pH of 2, with 100 $10^-6^ M of caffeine at a scan rate of 10 mV s^-1^ to 60 mV s^-1^

**Determination of caffeine in energy drink and pharmaceutical sample**

Determination of caffeine in energy drink and pharmaceutical sample is presented in Table S1.

**Table S1**. Determination of caffeine in energy drink and pharmaceutical sample

| **Sample** | **Amount found** | **RSD (%)** | **Standard added** | **Total found** | **Recovery (%)** |
| --- | --- | --- | --- | --- | --- |
| Imol plus | 24.4 mg/g | 2.5 | 25.0 mg/g | 49.56 | 101.9 |
| Ocean one’8 | 2.90 $ 10^-4^ mol L^-1^ | 2.9 | 3.00$10^-4^ mol L^-1^ | 4.53$10^-4^ mol L^-1^ | 94.9 |
